# Artificial Intelligence in Placental Pathology: New Diagnostic Imaging Tools in Evolution and in Perspective

**DOI:** 10.3390/jimaging11040110

**Published:** 2025-04-03

**Authors:** Antonio d’Amati, Giorgio Maria Baldini, Tommaso Difonzo, Angela Santoro, Miriam Dellino, Gerardo Cazzato, Antonio Malvasi, Antonella Vimercati, Leonardo Resta, Gian Franco Zannoni, Eliano Cascardi

**Affiliations:** 1 Pathology Unit, Department of Precision and Regenerative Medicine and Ionian Area (DiMePRe-J), University of Bari, Piazza Giulio Cesare 11, 70124 Bari, Italy; antonio.damati@uniba.it (A.d.); gerardo.cazzato@policlinico.ba.it (G.C.); leonardo.resta@policlinico.ba.it (L.R.); 2 Anatomic Pathology Unit, Fondazione Policlinico Universitario “A. Gemelli” IRCCS, Università Cattolica S. Cuore, 00136 Rome, Italy; angela.santoro@policlinicogemelli.it (A.S.); gianfranco.zannoni@unicatt.it (G.F.Z.); 3 1st Unit of Obstetrics and Gynecology, Department of Interdisciplinary Medicine (DIM), University of Bari, 70124 Bari, Italy; gbaldini97@gmail.com (G.M.B.); miriam.dellino@uniba.it (M.D.); antoniomalvasi@gmail.com (A.M.); antonella.vimercati@uniba.it (A.V.)

**Keywords:** digital pathology, artificial intelligence (AI), placental histopathology, machine learning (ML), pregnancy

## Abstract

Artificial intelligence (AI) has emerged as a transformative tool in placental pathology, offering novel diagnostic methods that promise to improve accuracy, reduce inter-observer variability, and positively impact pregnancy outcomes. The primary objective of this review is to summarize recent developments in AI applications tailored specifically to placental histopathology. Current AI-driven approaches include advanced digital image analysis, three-dimensional placental reconstruction, and deep learning models such as GestAltNet for precise gestational age estimation and automated identification of histological lesions, including decidual vasculopathy and maternal vascular malperfusion. Despite these advancements, significant challenges remain, notably dataset heterogeneity, interpretative limitations of current AI algorithms, and issues regarding model transparency. We critically address these limitations by proposing targeted solutions, such as augmenting training datasets with annotated artifacts, promoting explainable AI methods, and enhancing cross-institutional collaborations. Finally, we outline future research directions, emphasizing the refinement of AI algorithms for routine clinical integration and fostering interdisciplinary cooperation among pathologists, computational researchers, and clinical specialists.

## 1. Introduction

The placenta is a unique organ in two key ways: it is a temporary organ that undergoes continuous transformation throughout pregnancy, and it is the only organ that facilitates the connection between two living beings, providing nourishment, protection, and fostering fetal growth. Pathologists play a crucial role in identifying placental abnormalities that may explain or even predict adverse outcomes in subsequent pregnancies, such as metabolic or developmental disorders. In this context, histological evaluation of placental samples, combined with detailed clinical information, remains the gold standard for diagnosis [1,2].

However, in the era of precision medicine, there is an increasing need to reduce inter-observer variability and uncover valuable new data. Artificial intelligence (AI) has become an indispensable tool in this regard, offering significant potential for the investigation of placental diseases and enhancing the management of pregnancies [3,4]. It is no surprise that the first scientific studies in this field date back to the second half of the 20th century, and the literature now includes over 2000 publications on the topic [5].

Over the years, AI has been integrated with histochemical techniques such as confocal microscopy and immunohistochemistry. In recent years, AI and digital pathology have demonstrated numerous applications in anatomic pathology, including gynecologic pathology, where they have been tested for assessing MMR and PD-L1 status and predicting patient outcomes [6,7,8]. Recently, AI has also been used for 3D image analysis of the placenta, helping to assess not only the organ’s anatomical features but also morphological changes related to preeclampsia, gestational hypertension, trophoblastic disease, placental infections, accretions, and recurrent miscarriages [5,9]. In 2003, a study in Africa employed Kontron Systems 300 software to analyze trophoblast cells within the decidua and myometrium, demonstrating its potential to assess preeclampsia and its relationship to placental vascular remodeling [10]. Szilvia Szabo and colleagues also used digital systems to examine placental expression of syndecan-1 in preeclampsia, demonstrating an increase in immunoexpression in both early- and late-onset cases [11]. Additionally, semi-quantitative analysis has proven useful in evaluating inflammatory cells, such as decidual macrophages [12], and in assessing placental pathology in HIV+ patients [13]. These studies underscore the value of digital systems when combined with traditional evaluation methods in the study of placental accretions and intrauterine deaths. A more recent study using Image Pro Plus software focused on investigating the causes of fetal death, significantly reducing cases without a clear scientific explanation [14].

The main objective of this review is to summarize recent advancements in the application of artificial intelligence (AI) to placental pathology, emphasizing current diagnostic tools, promising methodologies, existing limitations, and future research perspectives. This review aims to provide pathologists and clinical researchers with a comprehensive understanding of how AI technologies can be integrated into routine placental diagnostics to improve accuracy, reproducibility, and clinical outcomes.

## 2. Brief Overview of AI, ML, and DL in Pathology

Artificial Intelligence (AI) encompasses computational systems designed to perform tasks that typically require human intelligence, such as pattern recognition and decision-making. Within AI, Machine Learning (ML) refers to algorithms that enable computers to learn from and make predictions or decisions based on data. A significant subset of ML is Deep Learning (DL), which utilizes artificial neural networks with multiple layers (hence “deep”) to model complex patterns in data. These neural networks are particularly adept at handling large, unstructured datasets, such as high-resolution histopathological images. In the realm of pathology, DL models—especially Convolutional Neural Networks (CNNs)—have been instrumental in advancing digital pathology. CNNs are designed to process and analyze visual data, making them ideal for interpreting histopathological slides. By learning hierarchical features from input images, CNNs can effectively identify patterns and anomalies that may be indicative of specific pathological conditions. This capability has been leveraged to develop AI tools that assist pathologists in diagnosing various diseases, including cancers and inflammatory conditions. For example, AI models have been developed to detect endometrial cancer with high accuracy by analyzing histopathological images, thereby aiding in early and precise diagnosis [15].

The integration of AI into pathology workflows offers several potential benefits:

- Enhanced Diagnostic Accuracy: AI algorithms can assist in identifying subtle histopathological features that may be challenging to detect with the naked eye, thereby reducing diagnostic errors.
- Increased Efficiency: Automated image analysis can expedite the diagnostic process, allowing pathologists to focus on more complex cases and potentially increasing overall productivity.
- Standardization: AI provides consistent analysis, minimizing inter-observer variability and contributing to more standardized diagnoses.

However, the implementation of AI in pathology also presents challenges:

- Data Quality and Quantity: Training robust AI models requires large datasets of high-quality, annotated images, which can be resource-intensive to compile.
- Interpretability: Many AI models, particularly deep learning networks, operate as “black boxes,” making it difficult to understand the rationale behind their predictions. This lack of transparency can hinder trust and acceptance among clinicians.
- Integration into Clinical Workflows: Incorporating AI tools into existing pathology practices necessitates careful consideration of workflow integration, user training, and regulatory compliance.

To address these challenges, ongoing research focuses on developing explainable AI models that provide insights into their decision-making processes, thereby enhancing their reliability and acceptance in clinical settings. Additionally, collaborative efforts are underway to create large, annotated datasets to improve the training and validation of AI algorithms in pathology [16,17]. 

Recent advances have also emphasized the importance of integrating explainable AI (XAI) with both handcrafted and deep learning-derived image features. For example, Rundo and Militello recently provided a comparative analysis of handcrafted features versus deep-learned features in medical imaging, highlighting how XAI can bridge the gap between model performance and clinical interpretability [18]. Their work reinforces the value of combining domain knowledge with data-driven methods to achieve both accuracy and transparency in diagnostic pipelines. Furthermore, Chou and Goldstein discussed how AI models, particularly those designed for placental imaging, can deepen our biological understanding of placental function and dysfunction. Their study highlights the dual utility of AI as both a diagnostic tool and a scientific discovery engine in placental biology [19].

## 3. Advancements in Artificial Intelligence for Placental Histology

Compared to previous reviews, our work specifically highlights recent innovations and emerging AI methods that have not been fully addressed elsewhere. Key novel contributions include discussion of state-of-the-art applications such as deep learning-based gestational age estimation (GestAltNet), 3D stereological reconstruction techniques, and CNN-driven automated classification of maternal vascular malperfusion (MVM) and decidual vasculopathy (DV), emphasizing their clinical implications.

Although AI has yet to be fully integrated into placental histopathological diagnostics, its rapid evolution is evident. Like a ball gaining momentum on a downhill slope, AI has found increasing applications in research. By harnessing the power of computational algorithms to emulate human learning and reasoning, AI systems are now able to distinguish more accurately between what is relevant and what is not. In some cases, they can even suggest possible courses of action. This development has revolutionized medicine in general and histopathological image analysis in particular, especially for the characterization of placental villi and vascular pathologies [20,21]. AI, through computerized morphometric analysis, allows for the precise evaluation of placental vessel diameters, the volume of placental villi, and comparisons between normal and pathological pregnancies, including cases with complications such as gestational diabetes [22] or recurrent miscarriages [23].

In 2021, Pooya Mobadersany and colleagues [24] introduced GestAltNet, a deep learning model designed to estimate the gestational age of villi from a simple histology scan with an average absolute error of approximately one week. This innovative approach has broad applications, from detecting villous maturation anomalies related to preeclampsia or gestational diabetes to offering automated analysis for recurrent abortions and impaired villous maturation. These advancements have not only expanded diagnostic capabilities but also contributed to the development of new AI techniques. GestAltNet employs a deep learning-based convolutional neural network with an attention mechanism to estimate gestational age from histological images, achieving an average absolute error of approximately one week. While this level of accuracy represents a significant advancement, its clinical impact should be interpreted with caution. Specifically, a one-week estimation error, though impressive, might still be clinically relevant depending on the gestational age and clinical context—particularly when diagnosing conditions sensitive to subtle variations, such as impaired villous maturation associated with preeclampsia or gestational diabetes. Additionally, the generalizability of GestAltNet remains uncertain, as the model was validated on a relatively homogeneous dataset. Future studies should address these limitations through comprehensive external validation across diverse populations and varied clinical settings. Similarly, recent innovations like “PlacentaVision”, which rapidly analyzes placental images to predict neonatal and maternal complications, show promise but also face methodological limitations. Such limitations include variability in image acquisition protocols and the potential for algorithmic biases arising from limited training datasets [25]. Hence, rigorous validation, standardized data collection protocols, and transparent model architectures are needed before widespread clinical adoption.

Recent advancements in machine learning (ML) have demonstrated significant potential in placental pathology. For instance, a study applied ML feature extraction methods to classify digital images of placental histopathology specimens, achieving promising results in detecting microscopic features indicative of adverse pregnancy outcomes. Additionally, data augmentation techniques, such as random cropping, have been employed to enhance the robustness of convolutional neural networks (CNNs) in medical image analysis, leading to improved model performance [26]. Furthermore, the application of digital imaging and artificial intelligence to placental pathology has been systematically reviewed, highlighting the integration of AI in diagnosing and understanding placental diseases [5]. Building upon these achievements, future studies could further unlock ML’s potential through improvements such as incorporating advanced data augmentation strategies to enhance the detection of partially visible structures and utilizing cross-institutional data to validate model generalizability.

The next frontier in AI for placental histopathology lies in the ability to analyze the organ in 3D. Automated systems are now capable of quantifying placental morphological features from histological slides, making it possible to examine larger sample areas and reduce variability between pathologists. Using stereological techniques, Saghi Zafaranieh and colleagues developed methods to analyze the volume of villi, intervillar spaces, vascular structures, and their densities, as well as fibrin areas and the relationships between various histological elements [27]. This 3D technology has significant potential for various applications, such as enhancing our understanding of placental physiology and providing insight into conditions like gestational diabetes [28], infections [29], and other placental diseases [30,31,32].

The ability to reconstruct the placenta in 3D by labeling serial histological slides has opened new possibilities. A recent English study developed a method to reconstruct an entire placenta using 200 consecutive 5 μm sections, enabling a comprehensive examination of histological architecture, identifying morphological differences in complicated pregnancies, and providing clinically relevant insights [33]. This approach may represent a major milestone in precision medicine, allowing for more accurate clinical decision-making for pregnant women.

Recent developments have significantly advanced AI-driven techniques in placental pathology. A notable example is “PlacentaVision”, an AI-powered tool designed to rapidly analyze placental images at birth to detect pathological abnormalities linked to neonatal infections and maternal complications, offering quicker diagnoses than conventional methods [25]. Another innovative technology, “HAPPY” (Histopathology Analysis Pipeline for Placental biopsY), integrates AI to precisely predict placental cell types and cell interactions from histological images, substantially enhancing our understanding of placental health and disease [34].

For these advancements to reach their full potential, it is crucial to recognize the importance of the pathologist’s role in this progression. Despite the impressive capabilities of AI, the constant training of algorithms and the interpretation of digital slides remain essential in refining these technologies for clinical use. Table 1 summarizes recent AI innovations in placental pathology, highlighting key applications, their methodological features, and clinical implications.

## 4. The Potential of Machine Learning in Placental Pathology

The application of AI-driven diagnostic tools has shown considerable promise in placental histopathology. For instance, a recent study successfully developed a computational pathology model capable of accurately identifying placental changes associated with preeclampsia, demonstrating high diagnostic precision and strong predictive capabilities [35]. Similarly, recent work has effectively utilized machine learning methods to classify digital histopathological images, automating the microscopic identification of placental lesions associated with maternal vascular malperfusion, thus providing consistent and objective diagnoses [26].

Success in developing machine learning (ML) models depends heavily on access to robust training datasets. Current research indicates preliminary success with supervised machine learning in placental pathology. For instance, models trained on 500–10,000 whole-slide images (WSIs) have accurately classified placental lesions [36,37,38]. Building on these achievements, future studies could further unlock ML’s potential through improvements such as (1) incorporating random cropping to enhance the detection of partially visible structures; and (2) utilizing cross-institutional data to validate model generalizability. Continued work is essential to improve traditional ML models and explore the full potential of deep learning in this domain. The integration of ML algorithms into placental histopathology analysis has already shown promising results [39].

There are several potential improvements and future directions for the proposed ML framework aimed at automating the inspection of placental lesions. In cancer pathology, random cropping has been used as a data augmentation technique to improve model detection of partially visible tumor structures. This approach may similarly apply to placental pathology, enhancing the identification of vascular lesions and parenchymal abnormalities [40,41,42]. For example, adding random cropping to the augmentation process during training could help simulate partially visible blood vessels and parenchymal lesions, making the model more robust to variations in normal histology. Additionally, analyzing image regions with artifacts, such as bubbles or tissue folds, could enhance the model’s ability to recognize and classify these common artifacts, improving prediction quality [43]. Future research could benefit from cross-institutional data to validate the generalizability of the algorithm. Recent studies suggest that regularized convolutional neural networks trained on single-institution data can be resilient to cohort variations when validated on data from other institutions [44].

Improvements in how output is presented to users—such as providing a gallery of high-resolution image patches sorted by confidence level—could boost explainability, confidence, and efficiency in analysis, especially for users who may not be familiar with AI. Future studies could also compare the current methods with those using only clinical metadata to determine which types of cases benefit most from WSI analysis. Previous research emphasizes the importance of accurately diagnosing placental parenchymal lesions, including infarcts, intervillous thrombi, and perivillous fibrin deposition, and highlights the potential of ML and digital pathology to improve diagnostic accuracy [44].

ML and digital pathology show significant promise in reducing interobserver variability and delivering expert-level examinations, even in settings with limited perinatal pathology expertise. They can assist in the rapid and uniform diagnosis of placental lesions, allowing for a more efficient screening process. Whole-slide learning, where the model analyzes the entire image, is a sophisticated approach but also presents challenges due to the data heterogeneity across a whole slide. Even normal slides contain multiple tissue types, complicating accurate lesion detection.

When discrepancies arise between human and AI diagnoses, it is crucial to have methods in place to evaluate and understand AI predictions for model improvement and post-deployment reasoning. Overall, ML and digital pathology present promising opportunities for enhancing the diagnosis of placental lesions, potentially enabling more efficient screening with fewer pathologists [45]. Partial automation of placental analysis through ML algorithms could provide microscopic placental analysis services to more mothers and infants, leading to more efficient inspections and standardized diagnoses. This could save lives in future pregnancies and reduce healthcare costs.

The development of robust AI models in placental pathology is fundamentally reliant on high-quality, annotated datasets. While several studies have reported promising models, the reproducibility and scalability of these tools remain limited due to the lack of standardized and publicly accessible data. Table 2 summarizes major datasets that have been used in the field, detailing their accessibility, type of data, annotations, and applicability to machine learning applications.

Although these datasets represent important steps forward, most are institution-specific and are not publicly accessible, limiting cross-institutional validation and algorithm generalizability. Future efforts should prioritize the creation of large, standardized, and open-access repositories of placental whole-slide images, ideally including detailed clinical metadata. Such initiatives would promote equity in AI development and facilitate robust benchmarking of novel diagnostic tools across different populations and clinical contexts.

## 5. Current Applications of AI and ML in Placental Histopathology

### 5.1. Decidual Vasculopathy

Decidual vasculopathy (DV), characterized by pathological alterations in the maternal decidual arteries, is a pivotal lesion associated with adverse pregnancy outcomes such as preeclampsia and fetal growth restriction. Traditional histopathological evaluation of DV is labor-intensive and subject to inter-observer variability. Recent advancements in artificial intelligence (AI) and digital pathology have sought to address these challenges by automating the detection and analysis of DV lesions. For instance, Clymer et al. developed a hierarchical machine learning approach utilizing multiresolution convolutional neural networks to automatically detect and classify DV lesions in digitized whole-slide images of placental tissues. This method demonstrated high accuracy in identifying DV, thereby facilitating large-scale screening and potentially enabling timely interventions to prevent conditions like preeclampsia [20]. Furthermore, the integration of AI with digital pathology platforms has enhanced the efficiency of placental examinations. The application of deep learning algorithms to whole-slide images has improved the identification of subtle histopathological features indicative of DV, thereby reducing diagnostic variability and enhancing reproducibility [35,46]. These advancements underscore the transformative potential of AI in standardizing and enhancing the diagnostic accuracy of DV in placental pathology.

Furthermore, recent research aimed to aggregate predictions from localized blood vessels to create a single data vector for disease classification at the image level, with a particular focus on detecting decidual vasculopathy (DV) within digital slides. DV lesions are characterized by abnormalities in decidual arterioles, such as fibrinoid necrosis of vessel walls, hypertrophy of the media, subendothelial lipid-laden macrophages, and possible thrombi in the lumen. The application of deep learning to analyze placental histopathology images holds significant promise for the early microscopic detection of DV lesions, which can aid in predicting preeclampsia risk in future pregnancies and enable preventive measures [20].

The deep learning pipeline employed in this research comprises three stages: object detection, classification, and aggregation. Two independently trained neural networks are used for object detection and classification, with a final aggregation step to combine the results. During object detection, the whole-slide image (WSI) is divided into a grid of patches, and each patch is analyzed for blood vessels. Bounding boxes with probabilities are generated for each patch, and higher-resolution patches are extracted from these locations for blood vessel classification. The classification stage then labels each blood vessel as either diseased or healthy. Histologic samples were taken from multiple regions of the placenta, including the umbilical cord, placental disc, and fetal membranes [47]. Digital images of the membranes, which are tightly rolled and cross-sectioned, were analyzed to focus on large cross-sectional areas of the decidual region, where DV lesions are most likely to occur [2].

To maximize classification accuracy, the network incorporates information from multiple regions of the image, rather than relying on features from a single blood vessel. Although the model is trained to detect blood vessels without distinguishing between healthy and diseased types during this stage, the primary focus is on the network’s ability to capture disease cases. The overarching goal is to identify DV within the digital slide.

At the whole-slide classification level, one diseased and one healthy case were misclassified. However, in the combined latent feature and patient metadata analysis, only one healthy case was misclassified. By aggregating blood vessel predictions and labeling the highest activated features from classified blood vessels, the algorithm reduces the risk of misclassification. This design was inspired by feedback from a perinatal pathologist during shadowing sessions, which highlighted that accurate DV classification could be achieved by observing DV in just a few blood vessels, even if most vessels were uncertain in their classification [20].

Blood vessel classification results demonstrated higher accuracy compared to the combined object detection and classification pipeline. A significant source of error in the combined ML pipeline appeared to stem from partially truncated blood vessels during the object detection stage. One potential solution would be to incorporate random crops into the augmentation pipeline during training to simulate partially visible blood vessels. Overall, this process emphasizes the challenges and considerations involved in aggregating predictions from localized blood vessels to classify disease at the image level, with a focus on improving accuracy and reducing sources of error. Table 3 summarizes the key features of AI-based detection of DV.

### 5.2. Maternal Vascular Malperfusion

**Figure 1:**
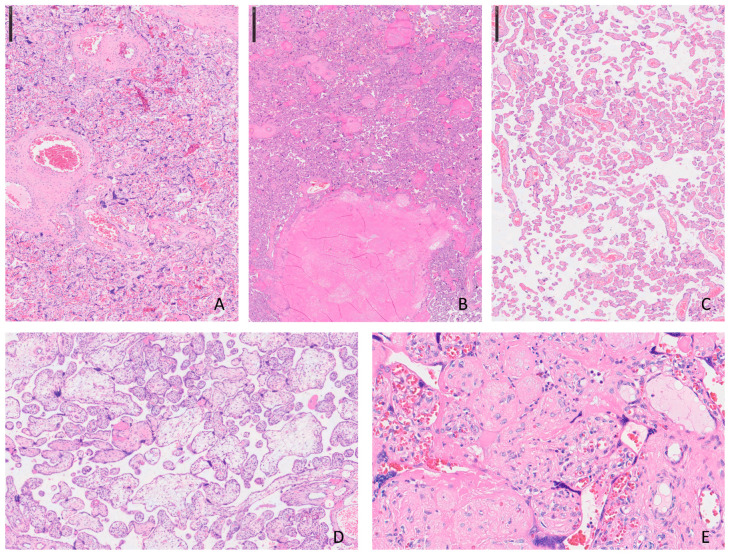
Histopathological features of maternal vascular malperfusion (MVM) in the placenta (H&E stain): (**A**) Collapsed chorionic villi with increased syncytial knotting, consistent with chronic hypoxia in the setting of MVM. (**B**) Area of villous infarction showing coagulative necrosis. (**C**) Distal villous hypoplasia with reduced number and size of terminal villi. (**D**) Accelerated villous maturation with numerous small, fibrotic terminal villi and increased syncytial knotting. (**E**) Increased perivillous fibrin deposition surrounding chorionic villi, partially entrapping them and impairing maternal–fetal exchange.

The placenta is a temporary organ formed during human pregnancy, responsible for transferring essential substances and waste between the mother’s and fetus’s bloodstreams. When placental health is compromised, it can lead to a range of conditions, with common obstetric complications such as hypertensive disorders of pregnancy (HDP) and fetal growth restriction (FGR). While the pathophysiology of these conditions is not yet fully understood, compromised utero-placental blood flow is observed in more than half of cases, often presenting as maternal vascular malperfusion (MVM) [44]. MVM is diagnosed through specific pathological features identifiable under microscopic examination, including decidual arteriopathy, placental infarcts, distal villous hypoplasia (DVH), and accelerated villous maturation. Typically, a detailed macro–microscopic examination reveals multiple co-existing pathological conditions affecting both the maternal and villous portions of the placenta (Figure 1) [48].

The illustration encloses the most frequent histological aspects observed in placental with MVM diagnosis. The photos come from slides stained with Hematoxylin and Eosin and scanned using digital pathology tools: in A can be observed an accelerated maturation of the placental villi and an increase in syncytial nodes (H.E. ×2.5); in B, miniature of placental infarction where groups of villi are associated with necrosis and loss of morphological details such as nuclear ones (H.E. ×2.5); a typical image of delayed maturation of the villi is represented in C (H.E. ×5); in D, two distinct populations of villi are depicted and the hydropic ones have larger dimensions and irregular profiles (H.E. ×2.5); and dense, pink material is the main characteristic of intervillous fibrin deposits seen in E (H.E. ×40).

Vascular or inflammatory issues within the villi can cause localized macroscopic lesions, necrotic material replacement, and adverse pregnancy outcomes. Histological analysis is crucial in identifying women who may require surgical intervention during delivery to prevent complications in future pregnancies, fetal malformations, and stillbirths [49].

Despite the increasing trend toward computerization and digitalization in pathology, placental diagnostic pathology remains an area that could greatly benefit from computer-aided diagnosis. Models have been developed to identify and assess various placental lesions and quantify the presence and severity of DVH using fractal dimensions. Additionally, convolutional neural network (CNN) regression models have been employed to automate the scoring of DVH and classify villous infarctions, perivillous fibrin deposition, and intervillous thrombus [44]. The advantages of AI and ML in this field are clear, as machine learning algorithms capable of recognizing visual patterns in placental histopathology specimens can significantly reduce inter-observer variability.

Patnaik et al. applied ML feature extraction methods to classify digital images of placental histopathology specimens, focusing on the presence or absence of MVM lesions. Their support vector machine classifier model achieved notable accuracy, demonstrating the potential of AI in automating the detection of MVM features [24]. Additionally, the application of deep learning models to whole-slide images has facilitated the automated identification of histopathological features associated with MVM, such as accelerated villous maturation and distal villous hypoplasia. These AI-driven approaches not only enhance diagnostic accuracy but also enable large-scale studies to elucidate the clinical implications of MVM in various pregnancy complications. The integration of AI into digital pathology workflows represents a significant advancement toward objective and reproducible assessments of MVM, ultimately contributing to improved maternal and fetal outcomes [9,35,50].

In 2023, artificial intelligence was used to develop a supervised ML model for classifying digital images of placental specimens based on the presence or absence of histological features associated with MVM. This study, conducted by the University of Ottawa, used a pre-trained ResNet18 model for feature extraction and demonstrated promising classification performance [51]. Under optimal testing conditions, which included data shuffling, augmentation, and classification at the whole-slide image (WSI) level using majority voting, the model achieved a classification accuracy of 79%. Specimens from patients with HDP and FGR were selected, as these conditions are commonly associated with MVM pathology [26]. The inclusion criteria for these groups were clearly defined, with HDP and FGR diagnosed based on gestational age, blood pressure, fetal weight, and umbilical artery Doppler findings. Healthy controls had no pre-existing or obstetrical health conditions. Each digital image was histopathologically examined by highly trained perinatal pathologists, who scored the images for the presence, absence, and severity of MVM-related lesions, including placental infarcts, DVH, accelerated villous maturation, and decidual arteriopathy. A cumulative MVM-feature score was assigned to each WSI, with scores ≥ 3 classified as MVM-positive (MVM+) and scores between 0 and 2 classified as MVM-negative (MVM−).

The performance of these models must consider the heterogeneity of histological features across the placenta. Some MVM lesions are diffusely spread, making them visible across both the original WSI and the image patches generated during model training. Other lesions, such as Accelerated Villous Maturation, tend to be more localized, labeling congested villi and thin terminal villi with increased intervillous space [48]. The model showed a tendency to misclassify certain patterns, such as interpreting the thinned-out villous tissue at the periphery of biopsies as artificial DVH. The poorest classification performance occurred on congested villous tissue areas within the accelerated villous maturation pattern, where the model misclassified them as MVM-negative. This suggests a bias in the model toward classifying DVH-like patterns as MVM-positive while incorrectly labeling focal villous clusters in accelerated villous maturation as MVM-negative. While poor utero-placental perfusion is considered a key component in the pathophysiology of HDP and FGR, not all cases show MVM evidence. Other mechanisms, such as pro-inflammatory processes or maternal predisposing factors, may contribute to these outcomes. Co-existing placental lesions, like villitis of unknown etiology or fetal vascular malperfusion, can distort MVM-feature extraction, limiting classification performance. FGR cases, both normotensive and hypertensive, demonstrated the highest misclassification rates compared to healthy controls and other obstetrical groups, possibly due to the enrichment of co-occurring lesions in FGR cases. Misclassifications were more common in early preterm cases (delivery < 34 weeks), and significant differences in gestational age at delivery were noted across MVM diagnostic categories [52,53].

Despite aligning with obstetrical outcomes such as HDP and FGR in 99% of MVM+ cases, placental disease presents a spectrum of lesion presence and severity. More severe pathology typically carries greater risks for both mother and fetus. Therefore, the chosen threshold for MVM classification is subjective, and WSIs with scores near this threshold are more likely to be misclassified [49]. Future work should address this limitation by comparing model performance at different MVM+ thresholds or developing multi-class models that better capture the spectrum of clinically observed pathology. Table 4 summarizes the key features of AI-based detection of MVM.

## 6. Challenges and Limitations of AI in Placental Pathology

### 6.1. Structural and Interpretative Limitations

The development of new artificial intelligence (AI) tools has undoubtedly propelled modern research, providing efficient and reproducible support in the diagnostic field. However, it is essential to recognize that these technologies are built upon specialized knowledge—knowledge that not only spans centuries of scientific development but also enables digital learning and adaptation. This understanding allows us to clearly identify the limitations of AI in placental histology, which are of a dual nature: structural and interpretative.

Structural limitations primarily stem from the inherent defects that may exist in histological slides, such as foreign material unintentionally incorporated into the tissue sample. In these cases, AI models may not be trained to detect or account for such anomalies, potentially leading to misinterpretations. For example, foreign objects mistakenly embedded in the slide could lead to incorrect conclusions, as AI might misclassify these artifacts as pathological features. Additionally, issues such as incomplete sections of tissue can complicate the analysis. When the AI encounters partial or fragmented sections, it might incorrectly classify them as lesions, such as distal villous hypoplasia, due to its reliance on patterns that it has learned during training. These structural challenges highlight the need for high-quality and artifact-free datasets to ensure that AI models can effectively discern between genuine pathological features and technical errors. A practical solution involves training AI models on augmented datasets enriched with annotated artifacts, enabling algorithms to differentiate artifacts from true pathology, as demonstrated by recent studies [54,55,56].

Interpretative limitations, on the other hand, arise from the AI’s ability—or inability—to fully understand the context of certain features in placental histology. For instance, AI systems might mistakenly identify intervillous fibrin deposits in peripheral histological samples, even though such deposits are not clinically significant or relevant for diagnosis. These kinds of errors reveal a fundamental challenge in AI: the difficulty in distinguishing between similar histological features that may have different clinical implications. Without a clear understanding of the broader clinical context, AI may inaccurately classify features, leading to false positives or misdiagnoses. This limitation can be addressed through hybrid approaches combining automated AI analysis with human expert validation. Explainable AI methods, such as SHAP or Grad-CAM, can further clarify algorithmic decision-making for pathologists [57,58].

These examples emphasize the importance of pathologist supervision in the AI-assisted diagnostic process. While AI can offer valuable support by automating the detection of lesions, it remains essential for pathologists to monitor the integrity and consistency of the data, ensuring that the identified lesions are pathologically significant and not due to artifacts or misinterpretations. The role of the pathologist is crucial not only for verifying the accuracy of the AI’s findings but also for differentiating between truly pathological features and reactive changes, which might otherwise mislead the AI model.

In addition to these limitations, there are broader challenges and obstacles that hinder the integration of AI into placental pathology. One major concern is the heterogeneity of placental tissues. The placenta is a highly diverse organ, with a variety of tissue types and pathological variations that can complicate AI analysis. Some lesions may be diffuse, while others are localized, making it difficult for AI systems to generalize across different cases. The variability of pathological features across specimens means that even sophisticated AI models may struggle to achieve consistent results. Furthermore, the complexity of interpreting placental histopathology images, which often involve subtle patterns and overlapping features, can lead to errors in classification. AI models may misinterpret or overlook critical features, such as small clusters of abnormal villi, especially when these features are not widely distributed throughout the placenta. Cross-institutional collaborations and the creation of standardized, publicly available datasets could significantly enhance dataset size and diversity, improving generalizability and reducing bias [55,57,58].

In fact, cross-institutional validation is critical to ensuring the generalizability and reliability of AI models in placental pathology. However, several significant challenges can limit successful validation. First, variability in histological staining protocols between institutions can significantly impact AI model performance, as differences in staining techniques introduce artifacts or alter tissue appearance, hindering the model’s ability to generalize across diverse datasets. Second, differences in scanning equipment and digital imaging parameters across laboratories may affect image resolution, contrast, and overall quality, further complicating AI model validation. Third, demographic and clinical variations across patient populations at different institutions may introduce biases, limiting AI model effectiveness across diverse groups. To overcome these challenges, researchers advocate for the development of standardized staining protocols, data normalization techniques, diverse training datasets, and rigorous validation frameworks that account for inter-institutional variability.

### 6.2. Dataset Challenges in AI Applications for Placental Pathology

Accurate annotation of placental histology images is crucial for training robust AI models. However, the complexity of placental structures and the subtlety of certain pathological features make manual annotation challenging and time-consuming. Additionally, inter-observer variability among pathologists can lead to inconsistent annotations, further complicating the training process. Developing standardized annotation guidelines and employing consensus approaches among experts can mitigate these issues [59].

Moreover, there is the issue of data quality. AI models require vast amounts of high-quality, annotated data to learn effectively. However, the availability of large, diverse datasets of placental histology images is limited, which poses a significant challenge in training AI systems that can generalize across different patient populations. Inadequate datasets can lead to biases in AI models, such as misclassifying certain pathologies or failing to recognize rare or complex lesions. Additionally, AI algorithms often function as “black boxes,” meaning their decision-making processes are not always transparent. This lack of interpretability can be a major limitation, as clinicians may be hesitant to rely on AI predictions without understanding the reasoning behind them. Implementing Explainable AI (XAI) approaches, such as attention maps or decision visualizations, will help clinicians better understand and trust AI-derived predictions, promoting smoother clinical integration [55,56].

Furthermore, AI models trained on datasets lacking diversity may not generalize well to broader populations. For instance, if a dataset predominantly includes samples from specific demographic groups, the resulting AI model may exhibit biased performance, leading to disparities in diagnostic outcomes. Ensuring that training datasets encompass a wide range of populations and pathologies is vital to developing equitable AI tools [60].

Another relevant issue is related to the histopathological analysis, which heavily relies on consistent staining techniques to highlight specific cellular components. However, variations in staining protocols across different laboratories can introduce artifacts, leading to inconsistencies in tissue appearance. These discrepancies pose significant challenges for AI models, which may misinterpret such artifacts as pathological features, thereby reducing diagnostic accuracy. Standardizing staining procedures is essential to minimize these variations and enhance the reliability of AI-driven analyses [61].

Despite considerable advances, significant challenges remain in effectively integrating AI into routine placental diagnostics. Inter-observer variability and the inherent complexity of histopathological images continue to necessitate more robust computational models. A recent 2024 study employed deep learning algorithms to detect maternal inflammatory response and histologic chorioamnionitis from whole-slide images of placental membranes, underscoring AI’s potential to elucidate complex inflammatory patterns [60]. Moreover, additional research utilizing AI to diagnose fetal inflammatory responses within umbilical cord tissues has further exemplified AI’s capability to automate challenging aspects of placental pathology assessment [62]. Such studies highlight ongoing efforts to overcome existing limitations and expand AI’s diagnostic potential in placental pathology.

### 6.3. Implementation Challenges for AI in Placental Pathology

(a) Regulatory Approval

Integrating AI into clinical settings necessitates navigating complex regulatory landscapes to ensure patient safety and efficacy. In the United States, the Food and Drug Administration (FDA) classifies AI-based diagnostic tools as medical devices, requiring rigorous evaluation before approval. Similarly, the European Union enforces the Medical Device Regulation (MDR) and the In Vitro Diagnostic Medical Devices Regulation (IVDR), which impose stringent standards for AI applications in healthcare. These regulatory frameworks can pose significant hurdles for the timely deployment of AI technologies in pathology [63,64].

(b) Cost and Infrastructure Requirements

The adoption of AI in pathology demands substantial financial investment in digital infrastructure. This includes acquiring high-resolution whole-slide imaging (WSI) scanners, establishing secure data storage solutions, and implementing high-performance computing systems capable of processing large datasets. Additionally, ongoing maintenance and technical support contribute to the overall costs, which may be prohibitive for smaller laboratories or institutions with limited resources [16,63].

(c) Pathologist Training and Acceptance

Successful implementation of AI tools hinges on the education and training of pathologists to effectively integrate these technologies into their workflows. Many pathologists may lack familiarity with AI methodologies, leading to resistance or apprehension toward adopting new tools. Comprehensive training programs are essential to equip pathologists with the necessary skills to interpret AI outputs and understand the limitations of these systems, thereby fostering confidence and acceptance [16,63].

### 6.4. Ethical Considerations for AI in Placental Pathology

(a) Patient Data Privacy and Confidentiality

The utilization of AI in placental pathology necessitates access to extensive datasets comprising sensitive patient information. Ensuring the privacy and confidentiality of this data is paramount. Compliance with regulations such as the General Data Protection Regulation (GDPR) in Europe and the Health Insurance Portability and Accountability Act (HIPAA) in the United States is essential to protect patient data. Implementing robust data anonymization and encryption techniques is vital to prevent unauthorized access and potential breaches [59,65].

(b) Informed Consent

The integration of AI tools into clinical practice raises questions about informed consent. Patients must be adequately informed about the use of AI in their diagnostic processes and the extent to which AI contributes to clinical decisions. Transparent communication is necessary to maintain trust and uphold patient autonomy [65].

(c) Algorithmic Bias and Fairness

AI systems trained on biased datasets may inadvertently perpetuate existing healthcare disparities. For instance, if training data predominantly represent certain populations, the AI may perform suboptimally for underrepresented groups, leading to inequitable care. Addressing algorithmic bias is crucial to ensure fairness and prevent the exacerbation of health inequities [66].

(d) Accountability and Transparency

The “black box” nature of some AI algorithms poses challenges in understanding their decision-making processes. This opacity can hinder clinicians’ ability to trust and effectively utilize AI recommendations. Developing explainable AI models is essential to enhance transparency and ensure that healthcare professionals can interpret and validate AI-driven insights [65].

(e) Impact of AI Misdiagnosis

AI misdiagnoses can have significant clinical consequences. Establishing clear guidelines for the deployment of AI tools, including protocols for human oversight and intervention, is necessary to mitigate potential harms resulting from AI errors [59].

## 7. Conclusions

In conclusion, while AI holds great potential in enhancing placental pathology diagnosis, its current limitations must be carefully considered. The technology’s ability to accurately detect and classify placental lesions is hindered by both structural and interpretative challenges, such as artifacts in slides and difficulty in distinguishing between similar pathological features. These limitations underscore the necessity for human oversight by pathologists to ensure the integrity and accuracy of the diagnostic process. Moving forward, continued research, better-quality datasets, and improvements in model transparency will be critical for addressing these challenges and unlocking the full potential of AI in placental pathology.

## Figures and Tables

**Table 1:** Recent Innovations and Emerging AI Methods in Placental Pathology.

AI Method	Authors	Year	Main Application	Technical Features	Clinical Implications
GestAltNet	Mobadersany et al. [24]	2021	Gestational age estimation from histological images	Deep learning CNN with attention mechanism; approx. ±1 week error	Early detection of villous maturation anomalies, gestational diabetes, preeclampsia
3D Stereological Reconstruction	Zafaranieh et al. [27]	2023	Quantification of villous volume, intervillar spaces, vascular structures, and fibrin areas	Automated stereological methods analyzing serial histological slides	Enhanced diagnosis of gestational diabetes, infections, and placental diseases
3D Placental Reconstruction	McCarthy et al. [33]	2023	Full 3D reconstruction from 200 serial histological slides	Automated labeling and digital alignment of serial sections	Detailed morphological analysis; improved decision-making for complicated pregnancies
PlacentaVision	Pan et al [25].	2024	Rapid placental image analysis at birth	AI-driven pathology detection of neonatal infections and maternal complications	Accelerated clinical intervention; early diagnosis
HAPPY	Vanea et al. [34]	2024	Prediction of placental cell types and cellular interactions	AI-based histopathology analysis pipeline	Improved understanding of placental health, disease mechanisms, and cell biology

**Table 2:** Overview of publicly and privately available datasets relevant to AI development and validation in placental pathology. The table includes access modalities, types of data, annotation details, and key usage notes.

Dataset/Repository	Access	Data Type	Annotations Available	Notes
The Cancer Genome Atlas (TCGA)—Placenta subset	Public	Genomic + limited WSIs	Clinical and molecular metadata	Limited placenta cases; useful for AI benchmarking
PathLAKE (UK NHS)	By request	Whole-slide images (WSIs)	Clinical metadata, outcome data	Placental cases included in organ-level collections
HAPPY Dataset (Vanea et al., 2024) [34]	Private (upon request)	H&E WSIs	Cell type segmentation, spatial relationships	Used for deep learning of cell-tissue interactions
PlacentaVision Dataset (Pan et al., 2024) [25]	Private	Histological and clinical images	Neonatal/maternal outcome annotations	Multimodal dataset; not yet publicly available
Institutional datasets (e.g., Ottawa, Pittsburgh)	Private	WSIs	Expert-labeled histological lesions	Used in training ML models for DV/MVM

**Table 3:** AI-Based Detection of Decidual Vasculopathy: Summary Features.

Aspect	Description
Definition	Decidual vasculopathy (DV) involves abnormalities in decidual arterioles, including fibrinoid necrosis and hypertrophy.
AI Application	Deep learning models analyze digital placental slides to detect microscopic DV lesions and predict preeclampsia risk.
Deep Learning Pipeline	Three stages: object detection (locating blood vessels), classification (healthy vs. diseased), and aggregation of results.
Challenges in DV Detection	Issues with truncated blood vessels and distinguishing between similar histological features can affect accuracy.
Pathologist Involvement	AI models assist but require human validation to confirm lesions and avoid misinterpretation of artifacts.
Future Directions	Refinements in AI training, increased dataset diversity, and better integration of clinical metadata to improve accuracy.

**Table 4:** AI-Based Detection of Maternal Vascular Malperfusion (MVM): Summary Features.

Aspect	Description
Definition	Maternal Vascular Malperfusion (MVM) is a placental condition associated with hypertensive disorders and fetal growth restriction (FGR).
AI Application	Machine learning models classify placental lesions indicative of MVM, such as infarcts, villous hypoplasia, and arteriopathy.
Automated MVM Classification	A ResNet18-based ML model achieved 79% accuracy in identifying MVM in placental samples from hypertensive and FGR pregnancies.
Challenges in MVM Diagnosis	Histological variability and overlapping placental lesions can lead to misclassification, particularly in preterm cases.
Impact on Clinical Outcomes	AI-based MVM detection can aid in early diagnosis and risk stratification for pregnancy complications.
Future Directions	Enhancements in dataset diversity, multi-class models, and refined classification thresholds to improve diagnostic precision.

## References

[1] Curtin W.M., Krauss S., Metlay L.A., Katzman P.J. (2007). Pathologic examination of the placenta and observed practice. Obstet. Gynecol..

[2] Sills A., Steigman C., Ounpraseuth S.T., Odibo I., Sandlin A.T., Magann E.F. (2013). Pathologic examination of the placenta: Recommended versus observed practice in a university hospital. Int. J. Women’s Health.

[3] Giorgione V., Cavoretto P., Cormio G., Valsecchi L., Vimercati A., De Gennaro A., Rabaiotti E., Candiani M., Mangili G. (2017). Prenatal diagnosis of twin pregnancies with complete hydatidiform mole and coexistent normal fetus: A series of 13 cases. Gynecol. Obstet. Investig..

[4] Beck R., Malvasi A., Kuczkowski K., Marinelli E., Zaami S. (2019). Intrapartum sonography of fetal head in second stage of labor with neuraxial analgesia: A literature review and possible medicolegal aftermath. Eur. Rev. Med. Pharmacol. Sci..

[5] Marletta S., Pantanowitz L., Santonicco N., Caputo A., Bragantini E., Brunelli M., Girolami I., Eccher A. (2023). Application of digital imaging and artificial intelligence to pathology of the placenta. Pediatr. Dev. Pathol..

[6] Addante F., d’Amati A., Santoro A., Angelico G., Inzani F., Arciuolo D., Travaglino A., Raffone A., D’Alessandris N., Scaglione G. (2024). Mismatch Repair Deficiency as a Predictive and Prognostic Biomarker in Endometrial Cancer: A Review on Immunohistochemistry Staining Patterns and Clinical Implications. Int. J. Mol. Sci..

[7] Santoro A., Angelico G., Inzani F., Arciuolo D., d’Amati A., Addante F., Travaglino A., Scaglione G., D’Alessandris N., Valente M. (2024). The emerging and challenging role of PD-L1 in patients with gynecological cancers: An updating review with clinico-pathological considerations. Gynecol. Oncol..

[8] Etrusco A., Lagana A.S., Chiantera V., Mikus M., Arsalan H.M., d’Amati A., Vitagliano A., Cicinelli E., Favilli A., D’Amato A. (2024). Reproductive and Oncologic Outcomes in Young Women with Stage IA and Grade 2 Endometrial Carcinoma Undergoing Fertility-Sparing Treatment: A Systematic Review. Biomolecules.

[9] Kozai A.C., Skvarca L.B., Parks W.T., Lane A., Gibbs B.B., Catov J.M. (2024). A novel technique to estimate intravillous fetal vasculature on routine placenta histologic sections. Placenta.

[10] Naicker T., Khedun S.M., Moodley J., Pijnenborg R. (2003). Quantitative analysis of trophoblast invasion in preeclampsia. Acta Obstet. Gynecol. Scand..

[11] Szabo S., Xu Y., Romero R., Fule T., Karaszi K., Bhatti G., Varkonyi T., Varkonyi I., Krenacs T., Dong Z. (2013). Changes of placental syndecan-1 expression in preeclampsia and HELLP syndrome. Virchows Arch..

[12] Bhalla A., Stone P., Liddell H., Zanderigo A., Chamley L. (2006). Comparison of the expression of human leukocyte antigen (HLA)-G and HLA-E in women with normal pregnancy and those with recurrent miscarriage. Reproduction.

[13] Moodley M., Moodley J., Naicker T. (2020). Evaluation of placental chorionic villi histone 2A expression in HIV-infected women with pre-eclampsia. Eur. J. Obstet. Gynecol. Reprod. Biol..

[14] Ptacek I., Smith A., Garrod A., Bullough S., Bradley N., Batra G., Sibley C.P., Jones R.L., Brownbill P., Heazell A.E. (2016). Quantitative assessment of placental morphology may identify specific causes of stillbirth. BMC Clin. Pathol..

[15] Ahmed A.A., Abouzid M., Kaczmarek E. (2022). Deep Learning Approaches in Histopathology. Cancers.

[16] Shafi S., Parwani A.V. (2023). Artificial intelligence in diagnostic pathology. Diagn. Pathol..

[17] McGenity C., Clarke E.L., Jennings C., Matthews G., Cartlidge C., Freduah-Agyemang H., Stocken D.D., Treanor D. (2024). Artificial intelligence in digital pathology: A systematic review and meta-analysis of diagnostic test accuracy. NPJ Digit. Med..

[18] Rundo L., Militello C. (2024). Image biomarkers and explainable AI: Handcrafted features versus deep learned features. Eur. Radiol. Exp..

[19] Chou T., Goldstein J.A. (2025). How can artificial intelligence models advance placental biology?. Placenta.

[20] Clymer D., Kostadinov S., Catov J., Skvarca L., Pantanowitz L., Cagan J., LeDuc P. (2020). Decidual vasculopathy identification in whole slide images using multiresolution hierarchical convolutional neural networks. Am. J. Pathol..

[21] Kidron D., Vainer I., Fisher Y., Sharony R. (2017). Automated image analysis of placental villi and syncytial knots in histological sections. Placenta.

[22] Maly A., Goshen G., Sela J., Pinelis A., Stark M., Maly B. (2005). Histomorphometric study of placental villi vascular volume in toxemia and diabetes. Hum. Pathol..

[23] Swiderska-Chadaj Z., Markiewicz T., Koktysz R., Cierniak S. (2018). Image processing methods for the structural detection and gradation of placental villi. Comput. Biol. Med..

[24] Mobadersany P., Cooper L.A.D., Goldstein J.A. (2021). GestAltNet: Aggregation and attention to improve deep learning of gestational age from placental whole-slide images. Lab. Investig..

[25] Pan Y., Mehta M., Goldstein J.A., Ngonzi J., Bebell L.M., Roberts D.J., Carreon C.K., Gallagher K., Walker R.E., Gernand A.D. (2024). Cross-modal contrastive learning for unified placenta analysis using photographs. Patterns.

[26] Patnaik P., Khodaee A., Vasam G., Mukherjee A., Salsabili S., Ukwatta E., Grynspan D., Chan A.D.C., Bainbridge S. (2024). Automated detection of microscopic placental features indicative of maternal vascular malperfusion using machine learning. Placenta.

[27] Zafaranieh S., Kummer D., van Poppel M.N., Desoye G., Huppertz B. (2023). Automated stereological image analysis approach of the human placenta: Surface areas and vascularization. Placenta.

[28] Heidari Z., Mahmoudzadeh-Sagheb H., Sheibak N. (2018). Quantitative changes of extravillous trophoblast cells in heavy smoker mothers compared with healthy controls. Reprod. Fertil. Dev..

[29] Ahenkorah J., Tetteh-Quarcoo P.B., Nuamah M.A., Kwansa–Bentum B., Nuamah H.G., Hottor B., Korankye E., Torto M., Ntumy M., Addai F.K. (2019). The impact of Plasmodium infection on placental histomorphology: A stereological preliminary study. Infect. Dis. Obstet. Gynecol..

[30] Heidari Z., Sakhavar N., Mahmoudzadeh-Sagheb H., Ezazi-Bojnourdi T. (2015). Stereological analysis of human placenta in cases of placenta previa in comparison with normally implanted controls. J. Reprod. Infertil..

[31] Güven D., Altunkaynak B., Altun G., Alkan I., Kocak I. (2018). Histomorphometric changes in the placenta and umbilical cord during complications of pregnancy. Biotech. Histochem..

[32] Kurtoglu E., Altunkaynak B.Z., Aydin I., Ozdemir A.Z., Altun G., Kokcu A., Kaplan S. (2015). Role of vascular endothelial growth factor and placental growth factor expression on placenta structure in pre-eclamptic pregnancy. J. Obstet. Gynaecol. Res..

[33] McCarthy R., Orsi N., Treanor D., Moran O., Vernooij M., Magee D., Roberts N., Stahlschmidt J., Simpson N. (2016). Three-dimensional digital reconstruction of human placental villus architecture in normal and complicated pregnancies. Eur. J. Obstet. Gynecol. Reprod. Biol..

[34] Vanea C., Dzigurski J., Rukins V., Dodi O., Siigur S., Salumae L., Meir K., Parks W.T., Hochner-Celnikier D., Fraser A. (2024). Mapping cell-to-tissue graphs across human placenta histology whole slide images using deep learning with HAPPY. Nat. Commun..

[35] Jung Y.M., Park S., Ahn Y., Kim H., Kim E.N., Park H.E., Kim S.M., Kim B.J., Lee J., Park C.W. (2024). Identification of Preeclamptic Placenta in Whole Slide Images Using Artificial Intelligence Placenta Analysis. J. Korean Med. Sci..

[36] Madabhushi A., Lee G. (2016). Image analysis and machine learning in digital pathology: Challenges and opportunities. Med. Image Anal..

[37] Tizhoosh H.R., Pantanowitz L. (2018). Artificial intelligence and digital pathology: Challenges and opportunities. J. Pathol. Inform..

[38] Dellino M., Cerbone M., d’Amati A., Bochicchio M., Laganà A.S., Etrusco A., Malvasi A., Vitagliano A., Pinto V., Cicinelli E. (2024). Artificial Intelligence in Cervical Cancer Screening: Opportunities and Challenges. AI.

[39] Cartus A.R. (2023). Machine learning to study placental pathology: Risk of reification and other considerations. Paediatr. Perinat. Epidemiol..

[40] Hao R., Namdar K., Liu L., Haider M.A., Khalvati F. (2021). A Comprehensive Study of Data Augmentation Strategies for Prostate Cancer Detection in Diffusion-Weighted MRI Using Convolutional Neural Networks. J. Digit. Imaging.

[41] Wang J., Kim C.-S., Kwak J.T. Patch stitching data augmentation for cancer classification in pathology images. Proceedings of the Medical Imaging 2024: Digital and Computational Pathology.

[42] Yang R., Wang R., Deng Y., Jia X., Zhang H. (2020). Rethinking the random cropping data augmentation method used in the training of CNN-based SAR image ship detector. Remote Sens..

[43] Irmakci I., Nateghi R., Zhou R., Vescovo M., Saft M., Ross A.E., Yang X.J., Cooper L.A., Goldstein J.A. (2024). Tissue contamination challenges the credibility of machine learning models in real world digital pathology. Mod. Pathol..

[44] Goldstein J.A., Nateghi R., Irmakci I., Cooper L.A. (2023). Machine learning classification of placental villous infarction, perivillous fibrin deposition, and intervillous thrombus. Placenta.

[45] Salafia C.M., Vintzileos A.M. (1990). Why all placentas should be examined by a pathologist in 1990. Am. J. Obstet. Gynecol..

[46] Hutchinson J.C., Picarsic J., McGenity C., Treanor D., Williams B., Sebire N.J. (2025). Whole Slide Imaging, Artificial Intelligence, and Machine Learning in Pediatric and Perinatal Pathology: Current Status and Future Directions. Pediatr. Dev. Pathol..

[47] Spencer M.K., Khong T.Y. (2003). Conformity to guidelines for pathologic examination of the placenta: Rates of submission and listing of clinical indications. Arch. Pathol. Lab. Med..

[48] Khong T.Y., Mooney E.E., Ariel I., Balmus N.C., Boyd T.K., Brundler M.-A., Derricott H., Evans M.J., Faye-Petersen O.M., Gillan J.E. (2016). Sampling and definitions of placental lesions: Amsterdam placental workshop group consensus statement. Arch. Pathol. Lab. Med..

[49] Romero R., Kim Y.M., Pacora P., Kim C.J., Benshalom-Tirosh N., Jaiman S., Bhatti G., Kim J.-S., Qureshi F., Jacques S.M. (2018). The frequency and type of placental histologic lesions in term pregnancies with normal outcome. J. Perinat. Med..

[50] Zur R.L., McLaughlin K., Aalto L., Jiang Y., Huszti E., Parks W.T., Kingdom J.C. (2024). Phenotypes of maternal vascular malperfusion placental pathology and adverse pregnancy outcomes: A retrospective cohort study. BJOG.

[51] He K., Zhang X., Ren S., Sun J. Deep residual learning for image recognition. Proceedings of the IEEE Conference on Computer Vision and Pattern Recognition.

[52] Redline R.W., Ravishankar S. (2018). Fetal vascular malperfusion, an update. Apmis.

[53] McBride C.A., Bernstein I.M., Sybenga A.B., McLean K.C., Orfeo T., Bravo M.C. (2022). Placental maternal vascular malperfusion is associated with prepregnancy and early pregnancy maternal cardiovascular and thrombotic profiles. Reprod. Med..

[54] Homeyer A., Geissler C., Schwen L.O., Zakrzewski F., Evans T., Strohmenger K., Westphal M., Bulow R.D., Kargl M., Karjauv A. (2022). Recommendations on compiling test datasets for evaluating artificial intelligence solutions in pathology. Mod. Pathol..

[55] Asadi-Aghbolaghi M., Darbandsari A., Zhang A., Contreras-Sanz A., Boschman J., Ahmadvand P., Kobel M., Farnell D., Huntsman D.G., Churg A. (2024). Learning generalizable AI models for multi-center histopathology image classification. NPJ Precis. Oncol..

[56] Van Booven D.J., Chen C.-B., Kryvenko O., Punnen S., Sandoval V., Malpani S., Noman A., Ismael F., Briseño A., Wang Y. (2024). Synthetic histology images for training ai models: A novel approach to improve prostate cancer diagnosis. bioRxiv.

[57] Devnath L., Summons P., Luo S., Wang D., Shaukat K., Hameed I.A., Aljuaid H. (2022). Computer-Aided Diagnosis of Coal Workers’ Pneumoconiosis in Chest X-ray Radiographs Using Machine Learning: A Systematic Literature Review. Int. J. Environ. Res. Public Health.

[58] Simon B.D., Ozyoruk K.B., Gelikman D.G., Harmon S.A., Turkbey B. (2024). The future of multimodal artificial intelligence models for integrating imaging and clinical metadata: A narrative review. Diagn. Interv. Radiol..

[59] Hanna M.G., Pantanowitz L., Jackson B., Palmer O., Visweswaran S., Pantanowitz J., Deebajah M., Rashidi H.H. (2025). Ethical and Bias Considerations in Artificial Intelligence/Machine Learning. Mod. Pathol..

[60] Vaidya A., Chen R.J., Williamson D.F.K., Song A.H., Jaume G., Yang Y., Hartvigsen T., Dyer E.C., Lu M.Y., Lipkova J. (2024). Demographic bias in misdiagnosis by computational pathology models. Nat. Med..

[61] Dunn C., Brettle D., Hodgson C., Hughes R., Treanor D. (2025). An international study of stain variability in histopathology using qualitative and quantitative analysis. J. Pathol. Inform..

[62] Ayad M.A., Nateghi R., Sharma A., Chillrud L., Seesillapachai T., Cooper L.A., Goldstein J.A. (2024). Deep Learning for Fetal Inflammatory Response Diagnosis in the Umbilical Cord. arXiv.

[63] Reis-Filho J.S., Kather J.N. (2023). Overcoming the challenges to implementation of artificial intelligence in pathology. J. Natl. Cancer Inst..

[64] Cheng J.Y., Abel J.T., Balis U.G.J., McClintock D.S., Pantanowitz L. (2021). Challenges in the Development, Deployment, and Regulation of Artificial Intelligence in Anatomic Pathology. Am. J. Pathol..

[65] Chauhan C., Gullapalli R.R. (2021). Ethics of AI in Pathology: Current Paradigms and Emerging Issues. Am. J. Pathol..

[66] Pantanowitz L., Hanna M., Pantanowitz J., Lennerz J., Henricks W.H., Shen P., Quinn B., Bennet S., Rashidi H.H. (2024). Regulatory Aspects of Artificial Intelligence and Machine Learning. Mod. Pathol..

